# c-Abl Inhibition Exerts Symptomatic Antiparkinsonian Effects Through a Striatal Postsynaptic Mechanism

**DOI:** 10.3389/fphar.2018.01311

**Published:** 2018-11-16

**Authors:** Yu Zhou, Yukio Yamamura, Masatoshi Ogawa, Ryosuke Tsuji, Koichiro Tsuchiya, Jiro Kasahara, Satoshi Goto

**Affiliations:** ^1^Department of Neurodegenerative Disorders Research, Institute of Biomedical Sciences, Graduate School of Medical Sciences, Tokushima University, Tokushima, Japan; ^2^Department of Neurobiology and Therapeutics, Institute of Biomedical Sciences, Graduate School of Pharmaceutical Sciences, Tokushima University, Tokushima, Japan; ^3^Department of Medical Pharmacology, Institute of Biomedical Sciences, Graduate School of Pharmaceutical Sciences, Tokushima University, Tokushima, Japan

**Keywords:** c-Abl, cyclin-dependent kinase 5, DARPP-32, parkinsonism, striatum

## Abstract

Parkinson’s disease (PD) is caused by a progressive degeneration of nigral dopaminergic cells leading to striatal dopamine deficiency. From the perspective of antiparkinsonian drug mechanisms, pharmacologic treatment of PD can be divided into symptomatic and disease-modifying (neuroprotective) therapies. An increase in the level and activity of the Abelson non-receptor tyrosine kinase (c-Abl) has been identified in both human and mouse brains under PD conditions. In the last decade, it has been observed that the inhibition of c-Abl activity holds promise for protection against the degeneration of nigral dopaminergic cells in PD and thereby exerts antiparkinsonian effects. Accordingly, c-Abl inhibitors have been applied clinically as a disease-modifying therapeutic strategy for PD treatment. Moreover, in a series of studies, including that presented here, experimental evidence suggests that in a mouse model of parkinsonism induced by *N*-methyl-4-phenyl-1,2,3,6-tetrahydropyridine, c-Abl inhibition exerts an immediate effect improving motor impairments by normalizing altered activity in striatal postsynaptic signaling pathways mediated by Cdk5 (cyclin-dependent kinase 5) and DARPP-32 (dopamine- and cyclic AMP-regulated phosphoprotein 32 kDa). Based on this, we suggest that c-Abl inhibitors represent an ideal antiparkinsonian agent that has both disease-modifying and symptomatic effects. Future research is required to carefully evaluate the therapeutic efficacy and clinical challenges associated with applying c-Abl inhibitors to the treatment of PD.

## Introduction

c-Abl (Abl1), also known as the Abelson murine leukemia viral oncogene homolog 1, is a member of the Abl family of non-receptor tyrosine kinases (Colicelli, 2011). As c-Abl is highly conserved and ubiquitously expressed in multiple mammalian cellular and subcellular components, it likely plays a role in the regulation of a wide variety of biological processes (Wang, 2014; Khatri et al., 2016). In the brain, under both normal and pathological conditions, c-Abl tyrosine kinase activity is linked to diverse neuronal functions related to cellular signaling (Lee et al., 2008; Ko et al., 2010; Klein et al., 2011), synapse formation (Finn et al., 2003; Perez de Arce et al., 2010; Vargas et al., 2014), and neurogenesis (Moresco and Koleske, 2003; Woodring et al., 2003; Schlatterer et al., 2012).

Observations from the past decade (for a review see, Brahmachari et al., 2017) indicate that impaired activity of c-Abl is implicated in the pathogenesis of Parkinson’s disease (PD), a neurodegenerative disorder caused by the progressive loss of dopamine (DA)-producing cells in the substantia nigra (Lotharius and Brundin, 2002; Dauer and Przedborski, 2003; Lang and Espay, 2018). There is an increase in the level and activity of c-Abl in human and mouse brains under PD conditions, which is evidently found in both the substantia nigra and striatum (Ko et al., 2010; Imam et al., 2011, 2013; Hebron et al., 2013a; Brahmachari et al., 2016). Experimental evidence has demonstrated that, in PD conditions, over-activation of c-Abl might induce parkin dysfunction (Imam et al., 2011; Gonfloni et al., 2012; Dawson and Dawson, 2014), a-synuclein aggregation (Hebron et al., 2013a; Mahul-Mellier et al., 2014; Brahmachari et al., 2016), and impaired autophagy of toxic elements (Ertmer et al., 2007; Yogalingam and Pendergast, 2008; Xu et al., 2017), leading to the death of the nigral dopaminergic cells. Accordingly, systemic administration of c-Abl inhibitors (e.g., imatinib and nilotinib) may hold promise for protection against the degeneration of nigral dopaminergic cells in PD and further antiparkinsonian effects (Hebron et al., 2013a,b; Karuppagounder et al., 2014; Wu et al., 2016; Zhou et al., 2017; Abushouk et al., 2018). Because antiparkinsonian drugs available at present do not appear to prevent the progression of PD, clinical applications of c-Abl inhibitors as a future disease-modifying therapeutic strategy for PD have been challenged (Brundin et al., 2013; Lindholm et al., 2016; Pagan et al., 2016; Brahmachari et al., 2017; Lang and Espay, 2018).

However, in our past studies, we have also demonstrated that c-Abl inhibitors might modulate striatal phosphorylation of specific protein targets at the postsynaptic level, and, thereby, exert antiparkinsonian effects. Abnormal motor behaviors in PD result from striatal dysfunction due to an imbalance between dopamine and glutamate transmission, which have opposing physiological effects (Olanow and Tatton, 1999; Greengard, 2001). A key regulator of the integration of DA and glutamate signals is the dopamine- and cyclic AMP-regulated phosphoprotein 32kDa (DARPP-32) (Greengard, 2001); corticostriatal glutamate inputs activate cyclin-dependent kinase 5 (Cdk5), which inhibits postsynaptic DA signaling by phosphorylating DARPP-32 at Thr75 (Bibb et al., 1999). We previously reported that c-Abl inhibition normalized motor impairments in a mouse model of PD induced by *N*-methyl-4-phenyl-1,2,3,6-tetrahydropyridine (MPTP). In this model, activity of Cdk5 can be inhibited by reducing the phosphorylation of Cdk5 at Tyr15 (Cdk5-Tyr15), leading to a decreased phosphorylation of DARPP-32 at Thr75 (DARPP-32-Thr75) in the striatum (Yamamura et al., 2013; Tanabe et al., 2014). The present study highlights striatal postsynaptic mechanisms by which c-Abl inhibitors represent a symptomatic antiparkinsonian agent to alleviate motor symptoms. Here, we show the immediate effects of imatinib (STI-571), a first-generation c-Abl inhibitor, on striatal c-Abl/Cdk5/DARPP-32 signaling pathway and striatal motor behaviors in MPTP-treated mice.

## Materials and Methods

### Animals

Male C57BL/6 mice (Japan SLC, Shizuoka, Japan) aged 7–8 weeks were used. The mice were housed under a 12-h light/dark cycle with *ad libitum* access to food and tap water. All experimental procedures were approved by the Committee for Animal Experiments of Tokushima University.

### MPTP Administration

Mice received intraperitoneal (i.p.) injections of MPTP-HCl (20 mg/kg of free base; Sigma–Aldrich, St. Louis, MO, United States) dissolved in 0.9% saline, 4 times per day for 1 day in 2 h-intervals (Tanabe et al., 2014). Saline-treated control mice received equivalent volumes of 0.9% saline. Our previous work demonstrated that maximal degenerative effects of MPTP on the nigral dopaminergic cells were observed when examined 3 days following MPTP administration (Aoki et al., 2009).

### Levodopa Administration

Mice received a single i.p. injection of levodopa (2.5, 5, or 15 mg/kg of free base; Sigma–Aldrich) dissolved in 0.9% saline containing 0.5% carboxymethyl cellulose 3 days after administration of MPTP or saline. Vehicle-treated mice received an equivalent volume of 0.9% saline containing 0.5% carboxymethyl cellulose. They were pre-treated with a single i.p. injection of benserazide (12.5 mg/kg; Sigma–Aldrich) dissolved in 0.9% saline 20 min before administration of levodopa or saline.

### Imatinib Administration

Mice received a single i.p. injection of imatinib mesylate (10 or 25 mg/kg; LKT Laboratories, St. Paul, MN, United States) dissolved in 0.9% saline containing 10% dimethyl sulfoxide 3 days after the administration of MPTP or saline. Vehicle-treated mice received an equivalent volume of 0.9% saline containing 10% dimethyl sulfoxide.

### Behavioral Tests

The beam-walking test evaluates motor coordination and balance in rodents. The testing apparatus consists of a rough round horizontal beam (wood, 8-mm-diameter for test trials or 16-mm-diameter for training trials, 80 cm long) fixed 60 cm above a countertop, and a dark goal box (15 cm wide, 10 cm long, and 10 cm tall). Mice were trained to traverse the beam without stopping on the way for three consecutive days before MPTP administration. In test trials, mice were made to traverse the beam in the same manner. The traveling time from the start to the 50-cm point was recorded (trials were cut-off at 60 s).

The rota-rod test evaluates motor coordination and motor learning. The Rota-Rod Treadmill (Constant Speed Model, Ugo Basile, Varese, Italy) was used. On the day prior to the first training session, mice were habituated to the apparatus for 5 min. Mice were trained to run on the rota-rod for 10 min at 20 rpm without falling, twice a day for three consecutive days before MPTP administration. In the test trials, mice were made to run on rod at 28 rpm (trials were cut-off at 600 s). The latency to fall was recorded.

### High Performance Liquid Chromatography (HPLC) Analysis

Mice were sacrificed by cervical dislocation 30 min after administration of imatinib or vehicle. Striatal tissues and plasma were rapidly sampled on ice and kept at -80°C until use. They were homogenized by glycine buffer (100 μM) at pH 2.75. Further, an Oasis PRiME Lipophilic Balance extraction cartridge (Waters Corporation, Milford, MA, United States) was used to extract imatinib from the tissues (Miura et al., 2011). HPLC analysis was conducted using an 880-PU Intelligent HPLC pump equipped with an 875-UV Intelligent UV/Vis detector (Jasco, Tokyo, Japan). Chromatographic separation was achieved using a Unison UK-C18 column (100 mm × 4.6 mm, 3 μm) at a flow rate of 1 ml/min. The concentration of imatinib was then analyzed using water/methanol/triethylamine (54:45:1) with a pH adjusted to 4.80 ± 0.05 as the mobile phase. The detection wavelength was set to 260 nm, and the injection volume was 50.0 μl. The striatal penetration of imatinib was assessed by striatum-to-blood concentration ratios, according to the method described previously (Bihorel et al., 2007).

For quantification of striatal DA and its metabolites, tissue samples were homogenized in 500 μl of perchronic acid (50 nM). After adding 400 μl of perchronic acid (50 nM) and 100 μl isoproterenol (as an internal standard substance, 1 μg/ml), homogenates were incubated on ice for 30 min, then centrifuged at 2,500 rpm for 15 min. Extracted samples (50 μl) were quantified via HPLC with an electrochemical detector (Eicom, Kyoto, Japan). The concentrations of DA, 3,4-dihydroxy-phenylacetic acid (DOPAC), and homovanillic acid (HVA) were analyzed using octane sulfonic acid (1.064 mM), EDTA-2Na (0.013 mM), 15% methanol, and a 0.1 M sodium citrate-0.1M sodium acetate buffer (pH 3.5) as the mobile phase. Chromatographic separation was achieved using an Eicompak SC-5ODS column (3.0ID × 150 mm). Concentrations of DA, DOPAC, and HVA were expressed as μg/g of total tissue weight (Kadoguchi et al., 2014).

### Western-Blot Analysis

Mice were sacrificed by cervical dislocation 30 min after administration of levodopa, imatinib, or vehicle. Striatal tissues were then quickly sampled and frozen with liquid nitrogen. Striatal tissue samples were prepared according to the Laemmli’s method with slight modifications, as in our previous report (Tanabe et al., 2014). Each sample, after being standardized to contain the same amount of protein as other samples, was subjected to 10% sodium-dodecylsulfate polyacrylamide gel electrophoresis, followed by blotting onto a polyvinylidene fluoride membrane. The blotted membranes were then incubated with the desired primary antibodies. Antibodies against tyrosine hydroxylase (TH, 1:1000; Millipore, Billerica, MA, United States), dopamine transporter (DAT, 1:1,000; Chemicon International, Temecula, CA, United States), vesicle monoamine transporter 2 (VMAT2, 1:500, Santa Cruz Biotechnology, Santa Cruz, CA, United States), Cdk5-pTyr15 (1:1,000; Santa Cruz Biotechnology, Santa Cruz, CA, United States), Cdk5 (1:1,000; Cell Signaling, Danvers, MA, United States), DARPP-32-pThr75 (1:1,000; Cell Signaling), DARPP-32-pThr34 (1:1,000; Cell Signaling), DARPP-32 (1:1,000; Cell Signaling), c-Abl (1:1,000; Cell Signaling), and c-Abl-pTyr412 (1:1,000; Cell Signaling) were used. Anti-β-actin antibody (1:5,000; Sigma–Aldrich) was used for adjustments to ensure that equal amounts of protein were loaded into each well. The bound antibodies were detected by the enhanced chemiluminescence method using horseradish peroxidase-conjugated secondary antibodies. Gel images were captured using a lumino-imaging analyzer LAS-4000 (Fujifilm, Tokyo, Japan). Optical densities were evaluated using a computerized image analysis system (Dolphin-DOC; Kurabo, Osaka, Japan).

### Statistical Analysis

All experimental values were expressed as means ± SEM. Statistical significance was evaluated by one-way analysis of variance (ANOVA) followed by the Scheffe *post hoc* test for pairwise comparisons. The significance level was set to *P* < 0.05. All analysis were conducted in Stat View 5.0 (SAS Institute, Cary, NC, United States).

## Results and Discussion

### Striatal Penetration of Peripherally Administered Imatinib in Naïve Mice

**FIGURE 1:**
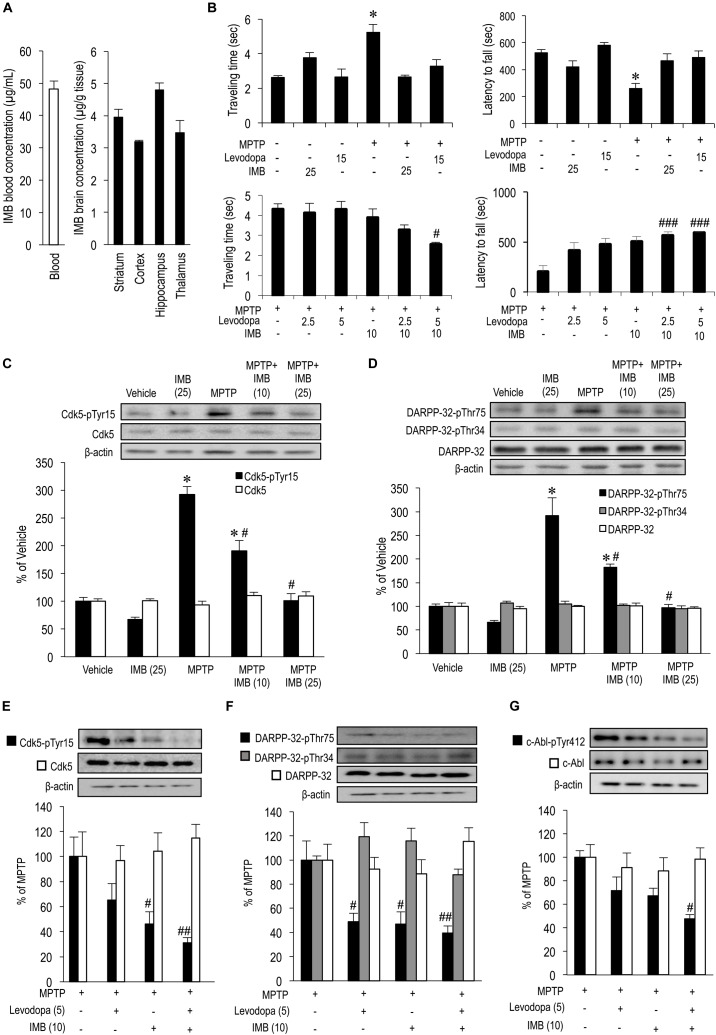
Effects of imatinib and levodopa on striatal motor behaviors and c-Abl/Cdk5/DARPP-32 signaling cascades. **(A)** Striatal penetration of intraperitoneally injected imatinib in mice. HPLC analysis were done to quantify concentrations of imatinib in the striatum (*n* = 4), cortex (*n* = 5), hippocampus (*n* = 4), thalamus (*n* = 4), and blood plasma (*n* = 5) of naïve mice that received single i.p. injections of imatinib mesylate (25 mg/kg) 30 min before sacrifice. Values are expressed as means ± SEM. **(B)** Symptomatic antiparkinsonian effects of imatinib and levodopa in MPTP-treated mice. Behavioral tests were carried out in vehicle or MPTP-treated mice 30 min after a single i.p. injection of imatinib and/or levodopa. (*left-upper panel*) The beam-walking test for examining the effects of administration of imatinib mesylate (25 mg/kg) or levodopa (15 mg/kg). Values are means ± SEM (*n* = 5–21). ^∗^*P* < 0.05 versus vehicle-treated mice; one-way ANOVA [*F*_(5,77)_ = 11.265] followed by the Scheffe *post hoc* test. (*right-upper panel*) The rota-rod test for examining the effects of imatinib mesylate (25 mg/kg) or levodopa (15 mg/kg) administration. Values are means ± SEM (*n* = 8–21). ^∗^*P* < 0.05 versus vehicle-treated mice; one-way ANOVA [*F*_(5,80)_ = 7.710] followed by the Scheffe *post hoc* test. (*left-lower panel*) The beam-walking test for examining the effects of imatinib mesylate (10 mg/kg) and/or levodopa (2.5 or 5 mg/kg) administration. Values are means ± SEM (*n* = 10–11). ^#^*P* < 0.05 versus MPTP-treated mice; one-way ANOVA [*F*_(5,55)_ = 4.177] followed by the Scheffe *post hoc* test. (*right-lower panel*) The rota-rod test for examining the effects of imatinib mesylate (10 mg/kg) and/or levodopa (2.5 or 5 mg/kg) administration. Values are means ± SEM (*n* = 10–11). ^###^*P* < 0.001 versus MPTP-treated mice; one-way ANOVA [*F*_(5,55)_ = 8.283] followed by the Scheffe *post hoc* test. **(C)** Western-blot analysis of striatal levels of Cdk5-pTyr15 and Cdk5 in vehicle or MPTP-treated mice 30 min after single i.p. injections of imatinib. Values are means ± SEM (*n* = 4–5). ^∗^*P* < 0.05 versus vehicle-treated mice, ^#^*P* < 0.05 versus MPTP-treated mice; one-way ANOVA [*F*_Cdk5-pTyr15(4,19)_ = 50.391, *F*_Cdk5(4,19)_ = 1.413] followed by the Scheffe *post hoc* test. IMB (10), imatinib mesylate (10 mg/kg); IMB (25), imatinib mesylate (25 mg/kg). **(D)** Western-blot analysis of striatal levels of DARPP-32-pThr75, DARPP-32-pThr34, and DARPP-32 in vehicle or MPTP-treated mice 30 min after a single i.p. injection of imatinib. Values are means ± SEM (*n* = 4–5). ^∗^*P* < 0.05 versus vehicle-treated mice, ^#^*P* < 0.05 versus MPTP-treated mice; one-way ANOVA [*F*_DARPP-32-pThr75(4,19)_ = 35.089, *F*_DARPP-32-pThr34(4,19)_ = 0.711, *F*_DARPP-32(4,19)_ = 0.293] followed by the Scheffe *post hoc* test. IMB (10), imatinib mesylate (10 mg/kg); IMB (25), imatinib mesylate (25 mg/kg). **(E)** Western-blot analysis of striatal levels of Cdk5-pTyr15 and Cdk5 in MPTP-treated mice 30 min after a single i.p. injection of levodopa and/or imatinib. Values are expressed as means ± SEM (*n* = 5–10). ^#^*P* < 0.05, ^##^*P* < 0.01 versus MPTP-treated mice. One-way ANOVA [*F*_Cdk5-pTyr15(3,31)_ = 6.039, *F*_Cdk5(3,17)_ = 0.258] followed by the Scheffe *post hoc* test. Levodopa (5), levodopa (5 mg/kg); IMB (10), imatinib mesylate (10 mg/kg). **(F)** Western-blot analysis of striatal levels of DARPP-32-pThr75, DARPP-32-pThr34, and DARPP-32 in MPTP-treated mice 30 min after a single i.p. injection of imatinib and/or levodopa. Values are expressed as means ± SEM (*n* = 4-10). ^#^*P* < 0.05, ^##^*P* < 0.01 versus MPTP-treated mice. One-way ANOVA [*F*_DARPP-32-pThr75(3,29)_ = 5.529, *F*_DARPP-32-pThr34(3,16)_ = 1.257, *F*_DARPP-32(3,16)_ = 2.886] followed by the Scheffe *post hoc* test. Levodopa (5), levodopa (5 mg/kg); IMB (10), imatinib mesylate (10 mg/kg). **(G)** Western-blot analysis of striatal levels of c-Abl-pTyr412, and c-Abl in MPTP-treated mice 30 min after a single i.p. injection of imatinib and/or levodopa. Values are expressed as means ± SEM (*n* = 8–11). ^#^*P* < 0.05 versus MPTP-treated mice; One-way ANOVA [*F*_c-Abl-pTyr412(3,34)_ = 5.820, *F*_c-Abl(3,29)_ = 0.240] followed by Scheffe *post hoc* test. Levodopa (5), levodopa (5 mg/kg); IMB (10), imatinib mesylate (10 mg/kg).

The blood-brain barrier (BBB), which contains various efflux transporters, represents a major obstacle to the delivery of most drugs to the central nervous system (Daneman and Prat, 2015). All c-Abl inhibitors have a limited BBB penetrance (Lindholm et al., 2016). Here, we used HPLC to quantify the concentrations of imatinib in the striatum, cortex, hippocampus, thalamus, and blood plasma from naïve mice that received a single i.p. injection of imatinib mesylate (25 mg/kg) 30 min before sacrifice (Figure 1A). Our results showed that the concentration of imatinib in the striatum was 3.961 ± 0.236 μg/g of tissue weight, in cortex was 3.198 ± 0.046 μg/g of tissue weight, in the hippocampus was 4.806 ± 0.221 μg/g of tissue weight, in the thalamus was 3.472 ± 0.382 μg/g of tissue weight, while that in the blood plasma was 48.23 ± 2.51 μg/ml. Accordingly, the striatum-to-blood concentration ratio of imatinib was 0.083 ± 0.005. Thus, peripherally administered imatinib was partially incorporated into the striatum, as had been suggested previously (Breedveld et al., 2005; Bihorel et al., 2007).

### Systemic Administration of Imatinib Normalizes Striatal Motor Behaviors in MPTP-Treated Mice

We previously reported that systemic administration of nilotinib (AMN107), a second-generation c-Abl inhibitor, exerted an immediate, therapeutic impact on motor deficits in MPTP-treated mice, as assessed by the beam-walking test, rota-rod test, bar test, horizontal-wire test, and foot-printing test (Tanabe et al., 2014). In this study, we also performed behavioral tests in MPTP-treated mice 30 min after administration of imatinib and/or levodopa. The beam-walking test revealed that, in MPTP-treated mice, an abnormal increase in the traveling time was significantly reversed by administration of imatinib mesylate (25 mg/kg) or levodopa (15 mg/kg) (Figure 1B, *left-upper panel*; ^∗^*P* < 0.05). The rota-rod test also revealed that, in MPTP-treated mice, the latency to fall was returned to normal levels after administration of imatinib mesylate (25 mg/kg) or levodopa (15 mg/kg) (Figure 1B, *right-upper panel*; ^∗^*P* < 0.05). It was also noted that, in MPTP-treated mice, administration of levodopa (2.5 or 5.0 mg/kg) or imatinib mesylate (10 mg/kg) alone had no effect on traveling time or latency to fall, as determined by the beam-walking (Figure 1B, *left-lower panel*) and rota-rod (Figure 1B, *right-lower panel*) tests. However, the beam-walking test revealed that administration of levodopa (5.0 mg/kg) combined with imatinib mesylate (10 mg/kg) significantly reduced the traveling time in MPTP-treated mice (Figure 1B, *left-lower panel*; ^#^*P* < 0.05). Moreover, the rota-rod test showed that administration of levodopa (2.5 or 5.0 mg/kg) combined with imatinib mesylate (10 mg/kg) significantly increased the latency to fall in MPTP-treated mice (Figure 1B, *right-lower panel*; ^###^*P* < 0.001). Collectively, these findings suggest that levodopa and imatinib synergistically improve motor deficits in MPTP-treated mice.

### Systemic Administration of Imatinib Normalizes Striatal c-Abl/Cdk5/DARPP-32 Signaling Cascades in MPTP-Treated Mice

We have previously demonstrated that the immediate therapeutic effects of c-Abl inhibitors are associated with a normalization of altered striatal Cdk5 and DARPP-32 signals in MPTP-treated mice (Yamamura et al., 2013; Tanabe et al., 2014). c-Abl is known to phosphorylate Cdk5-Tyr15 and thereby facilitates Cdk5 activity (Zukerberg et al., 2000; Dhavan and Tsai, 2001; Zhang et al., 2007). In normal mice, Cdk5-pTyr15 is highly enriched in the striatum (Morigaki et al., 2011), where dopaminergic stimulation inhibits phosphorylation of Cdk5-Tyr15 via the D_2_-type dopamine receptors (Yamamura et al., 2013). In the striatum of MPTP-treated mice, DA deficiency causes an increased phosphorylation of both Cdk5-Tyr15 and DARPP-32-Thr75, which is reversed by c-Abl inhibition (Yamamura et al., 2013).

Here, we reappraised the effects of c-Abl inhibition on striatal c-Abl/Cdk5/DARPP-32 signaling cascades in MPTP-treated mice 30 min after administration of imatinib and/or levodopa. Western-blot analysis revealed a significant increase in striatal levels of Cdk5-pTyr15 (Figure 1C; ^∗^*P* < 0.05) and DARPP-32-pThr75 (Figure 1D; ^∗^*P* < 0.05) in MPTP-treated mice as compared with vehicle-treated mice, which was reversed by the administration of imatinib mesylate in a dose dependent manner (10 and 25 mg/kg, Figures 1C,D; ^#^*P* < 0.05). It was also noted that in MPTP-treated mice, an abnormal increase in striatal levels of Cdk5-pTyr15 was significantly reduced by administration of imatinib mesylate (10 mg/kg) alone (Figures 1C,E; ^#^*P* < 0.05) or imatinib mesylate (10 mg/kg) combined with levodopa (5 mg/kg) (Figure 1E; ^##^*P* < 0.01). Administration of levodopa (5 mg/kg) alone had no effect on striatal levels of Cdk5-pTyr15 (Figure 1E). In parallel, an abnormal increase in striatal levels of DARPP-32-pThr75 was significantly reduced by administration of imatinib mesylate (10 mg/kg) alone (Figures 1D,F; ^#^*P* < 0.05), levodopa (5.0 mg/kg) alone, or imatinib mesylate (10 mg/kg) combined with levodopa (5.0 mg/kg) (Figure 1F; ^#^*P* < 0.05, ^##^*P* < 0.01). Administration of imatinib mesylate (10 mg/kg) alone, levodopa (5.0 mg/kg) alone, or imatinib mesylate (10 mg/kg) combined with levodopa (5.0 mg/kg) has no effect on striatal levels of DARPP-32-pThr34 (Figure 1F). Moreover, striatal levels of c-Abl-pTyr412, an active form of c-Abl (Brasher and Van Etten, 2000), were significantly reduced by the administration of imatinib mesylate (10 mg/kg) combined with levodopa (5.0 mg/kg) in MPTP-treated mice (Figure 1G; ^#^*P* < 0.01). Thus, imatinib showed an inhibitory effect on the Cdk5/DARPP-32-signaling pathway in the striatum, as did levodopa.

### No Effects of Imatinib on Striatal Presynaptic Dopaminergic Markers in MPTP-Treated Mice

**FIGURE 2:**
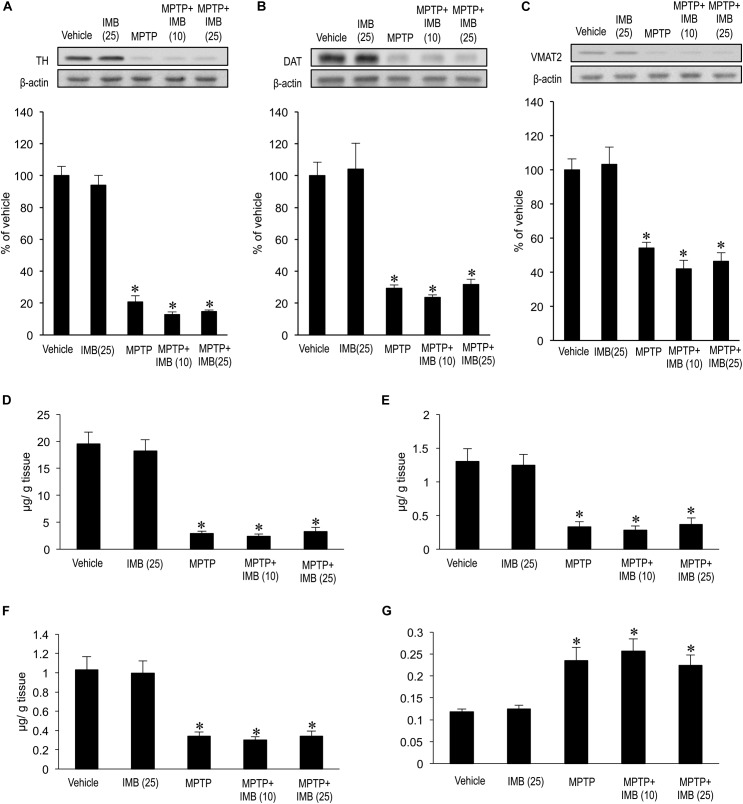
Effects of imatinib on striatal presynaptic dopaminergic markers in MPTP-treated mice. Western-blot and HPLC analyses were carried out on the striatal extracts from vehicle or MPTP-treated mice 30 min after a single i.p. injection of imatinib mesylate (10 or 25 mg/kg). **(A)** Western-blot analysis of striatal levels of TH. Values are means ± SEM (*n* = 4-5). ^∗^*P* < 0.05 versus vehicle-treated mice; one-way ANOVA [*F*_(4,19)_ = 107.43] followed by the Scheffe *post hoc* test. IMB (10), imatinib mesylate (10 mg/kg); IMB (25), imatinib mesylate (25 mg/kg). **(B)** Western-blot analysis of striatal levels of DAT. Values are means ± SEM (*n* = 4–5). ^∗^*P* < 0.05 versus vehicle-treated mice; one-way ANOVA [*F*_(4,19)_ = 21.749] followed by the Scheffe *post hoc* test. IMB (10), imatinib mesylate (10 mg/kg); IMB (25), imatinib mesylate (25 mg/kg). **(C)** Western-blot analysis of striatal levels of VMAT2. Values are means ± SEM (*n* = 4–5). ^∗^*P* < 0.05 versus vehicle-treated mice; one-way ANOVA [*F*_(4,19)_ = 20.615] followed by the Scheffe *post hoc* test. IMB (10), imatinib mesylate (10 mg/kg); IMB (25), imatinib mesylate (25 mg/kg). **(D–G)** HPLC analysis of striatal levels of DA **(D)**, DOPAC **(E)**, HVA **(F)**, and DA-turnover, which represents a net dopamine usage in striatum with (DOPAC + HVA)/DA **(G)**. Values are expressed as means ± SEM (*n* = 4–5). ^∗^*P* < 0.05 versus vehicle-treated mice; one-way ANOVA [*F*_DA(4,19)_ = 34.526, *F*_DOPAC(4,19)_ = 15.383, *F*_HV A(4,19)_ = 16.078, *F*_DA-turnover(4,19)_ = 10.355] followed by the Scheffe *post hoc* test. IMB (10), imatinib mesylate (10 mg/kg); IMB (25), imatinib mesylate (25 mg/kg).

We also examined the effects of c-Abl inhibition on striatal presynaptic dopaminergic markers in MPTP-treated mice 30 min after administration of imatinib. Western-blot analysis revealed that striatal levels of TH (Figure 2A, ^∗^*P* < 0.05), DAT (Figure 2B, ^∗^*P* < 0.05), and VMAT2 (Figure 2C, ^∗^*P* < 0.05) were significantly lower in the MPTP-treated mice than in the vehicle-treated mice. Notably, administration of imatinib mesylate (10 or 25 mg/kg) had no effects on striatal levels of TH, DAT, or VMAT2 in either normal or MPTP-treated mice (Figures 2A–C). HPLC analysis also revealed a significant reduction in striatal levels of DA (Figure 2D; ^∗^*P* < 0.05), DOPAC (Figure 2E; ^∗^*P* < 0.05), and HVA (Figure 2F; ^∗^*P* < 0.05) in MPTP-treated mice when compared to vehicle-treated mice. We found that administration of imatinib mesylate (10 or 25 mg/kg) had no effects on striatal levels of DA, DOPAC, or HVA in MPTP-treated mice, as in normal mice (Figures 2D–F). Striatal DA-turnover, as calculated by dividing the total amount of DOPAC and HVA by DA content, was significantly increased in MPTP-treated mice (Figure 2G; ^∗^*P* < 0.05) as compared to vehicle-treated mice. We found that administration of imatinib mesylate (10 or 25 mg/kg) had no effects on striatal DA-turnover in either normal or MPTP-treated mice (Figure 2G). Taken together, these results suggest that the immediate therapeutic effects of imatinib in MPTP-treated mice depend on postsynaptic, but not presynaptic, striatal mechanisms.

## Conclusion

**FIGURE 3:**
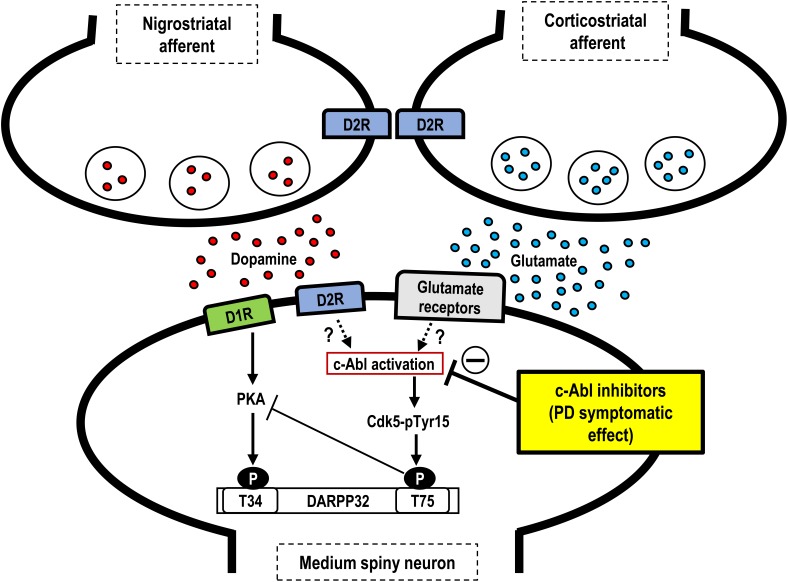
Hypothetical scheme showing symptomatic antiparkinsonian effects of c-Abl inhibitors. Depicted are the possible roles of c-Abl inhibitors at post-synaptic levels in the striatum. In the postsynaptic medium spiny neurons, dopamine deficiency may induce c-Abl activation to increase phosphorylation of Cdk5 at Tyr15 (Cdk5-Tyr15) and DARPP-32 at Thr75 (DARPP-32-Tyr75), resulting in an increased activity of the Cdk5/DARPP32-Thr75 pathway, which leads to parkinsonian symptoms. Thus, c-Abl inhibitors may exert symptomatic antiparkinsonian effects at the post-synaptic level. PD, Parkinson’s disease; D1R, D1-type dopamine receptor; D2R; D2-type dopamine receptor; Cdk5-pTyr15, Cdk5 with tyr15 phosphorylation; T34, Threonine 34; T75, Threonine 75.

Our results demonstrate that, in MPTP-treated mice, imatinib has a therapeutic effect on abnormal motor behaviors within an hour after its systemic administration. This symptomatic therapeutic action is associated with a normalization of otherwise increased phosphorylation of striatal Cdk5-Tyr15 and DARPP-32-Thr75, but has no effects on striatal presynaptic dopaminergic markers. As a major striatal postsynaptic mechanism, DA and glutamate signals have been demonstrated to play opposing physiological roles via a positive feedback loop that amplifies their mutually antagonistic actions (Greengard, 2001). Given this, glutamate inputs could activate Cdk5, leading to increased phosphorylation of DARPP-32-Thr75 and thereby antagonizing striatal DA functions (Bibb et al., 1999). Although the precise mechanism by which c-Abl activity involves interactions between DA and glutamate transmission in the striatum remains unknown, land mark reports demonstrated that c-Abl could phosphorylate Cdk5-Tyr15 to increase Cdk5 activity (Zukerberg et al., 2000; Zhang et al., 2007). Based on a series of studies conducted by our group, including the one presented here, we hypothesize that c-Abl inhibition exerts symptomatic antiparkinsonian actions via inhibition of phosphorylation of Cdk5-Tyr15, thereby, resulting in decreased activity of the striatal glutamate/Cdk5/DARPP-32 pathway (for reference see Figure 3). Further studies with targeting these molecules would clarify the consequence between them and PD-related motor symptoms. Together with additional intriguing evidence that c-Abl inhibition may exert disease-modifying effects on PD by playing a protective role against the degeneration of nigral dopaminergic cells (Ko et al., 2010; Imam et al., 2011, 2013; Hebron et al., 2013a,b), we suggest that c-Abl inhibitors may serve as an alternative agent for attenuating motor symptoms and disease progression in patients with PD. However, the c-Abl inhibitors currently approved for use in clinics have limited BBB penetrance (Lindholm et al., 2016), as demonstrated here for imatinib, which may limit their clinical utility. In addition, although some c-Abl inhibitors are widely used in the treatment of chronic myelocytic leukemia, the potential risk that they pose for causing serious systemic adverse effects has been reported (Arora and Scholar, 2005). The development of new therapeutic interventions to deliver c-Abl inhibitors to specific brain regions may be needed for their long-term use in the treatment of PD.

## Author Contributions

SG and JK participated in research design. YZ, YY, MO, RT, KT, JK, and SG conducted the experiments. KT, JK, and SG contributed new reagents or analytic tools. YZ, YY, KT, JK, and SG performed data analysis. SG, YZ, and JK wrote or contributed to the writing of the manuscript.

## Conflict of Interest Statement

The authors declare that the research was conducted in the absence of any commercial or financial relationships that could be construed as a potential conflict of interest.
